# Case Reports: A role of postoperative radiation therapy in completely resected early stage intrathyroid thymic carcinoma: a case report and literature review of the diagnosis and treatment

**DOI:** 10.3389/fonc.2023.1234961

**Published:** 2023-10-02

**Authors:** Ailin Cui, Yaoqiang Du, Chunjie Hou, Lin Zhang, Litao Sun, Hongfeng He

**Affiliations:** ^1^ Cancer Center, Department of Ultrasound Medicine, Zhejiang Provincial People’s Hospital (Affiliated People’s Hospital), Hangzhou Medical College, Hangzhou, Zhejiang, China; ^2^ Laboratory Medicine Center, Department of Transfusion Medicine, Zhejiang Provincial People’s Hospital (Affiliated People’s Hospital), Hangzhou Medical College, Hangzhou, Zhejiang, China; ^3^ Key Laboratory of Endocrine Gland Diseases of Zhejiang Province, Hangzhou, Zhejiang, China; ^4^ Department of Ultrasound, Hangzhou Children’s Hospital, Hangzhou Children’s Hospital of Wenzhou Medical University, Hangzhou, Zhejiang, China

**Keywords:** thyroid tumor, intrathyroidal thymic carcinoma, immunohistochemistry, surgery, radiotherapy

## Abstract

**Background:**

Intrathyroid thymic carcinoma (ITTC) is a rare malignant tumor of the thyroid, probably arising from ectopic thymus or branchial pouch remnants. Most of the literature recommended radical resection as the fundamental treatment for ITTC, and postoperative radiation appears to be able to reduce the recurrence rate in patients with advanced ITTC. However, the issue of adjuvant radiotherapy in completely resected early-stage ITTC has been controversial.

**Case presentation:**

Here, we reported a new case of early-stage ITTC that treated with total thyroidectomy and the right central neck dissection. Postoperative external beam radiation therapy (50.0 Gy/25 fractions) was given to the thyroid bed and bilateral cervical lymph node area since the tumor involved part of the sternal thyroid muscle. At 4-year follow-up after completion of radiotherapy, she is without evidence of locally recurrent or distant disease.

**Conclusion:**

Since there are no current guidelines for early-stage ITTC, in combination with this case and previous literature, we may suggest routine adjuvant radiotherapy should be considered in patients with incompletely resected tumors and extraparenchymal extension of ITTC. Moreover, we summarized comprehensive and advanced diagnosis, treatment, prognosis of ITTC and comparison between ITTC, primary squamous cell carcinoma of thyroid gland, differentiated thyroid cancer, and anaplastic thyroid cancer.

## Introduction

Intrathyroid thymic carcinoma (ITTC) is an extremely rare malignant neoplasm of the thyroid gland that grows slowly and has a low degree of aggressiveness. It is believed to originate from ectopic thymus tissue or remnants of branchial pouches. The term “intrathyroidal epithelial thymoma” was coined by Miyauchi et al. in 1985 to describe this type of cancer (1, 2). Histologically, it exhibits similarities to squamous-cell carcinoma (SCC) and anaplastic carcinoma (AC) of the thyroid. However, it is characterized by an indolent clinical course and favorable prognosis (3–6). Since its low incidence, there has been limited information available guiding the management of ITTC, particularly in its early stages. Furthermore, given the absence of typical imaging features, radiologists may misdiagnose it as other aggressive thyroid cancers causing clinical excessive treatments. In this study, we have reported a new case of ITTC and conducted a systematic review of other cases to aid in enhancing differential diagnosis and treatment strategies.

## Case report

**Figure 1:**
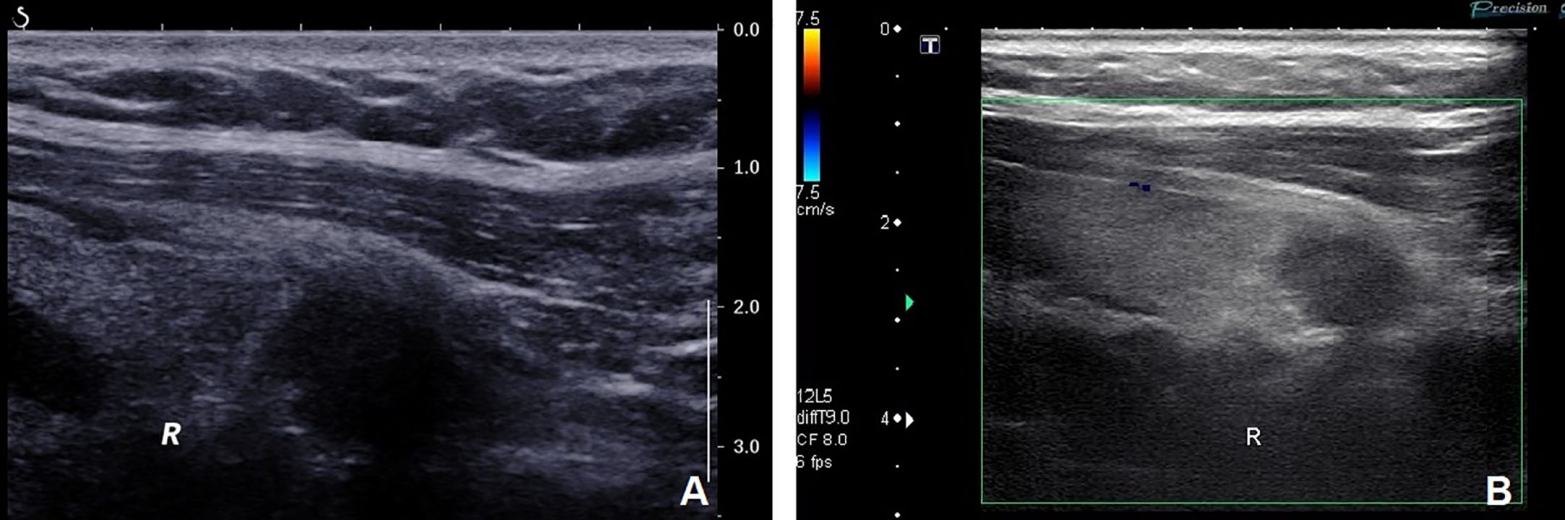
**(A)** Neck ultrasonography showed a solid hypoechoic nodule measuring 16mm×11mm×13mm in the middle-lower part of the right thyroid. The nodule exhibited irregular shape and unclear border but without calcification and linear hyperechoic patterns. The ratio of nodule longitudinal diameter to transverse diameter was less than 1. **(B)** Color Doppler sonography revealed scanty blood flow within the nodule.

**Figure 2:**
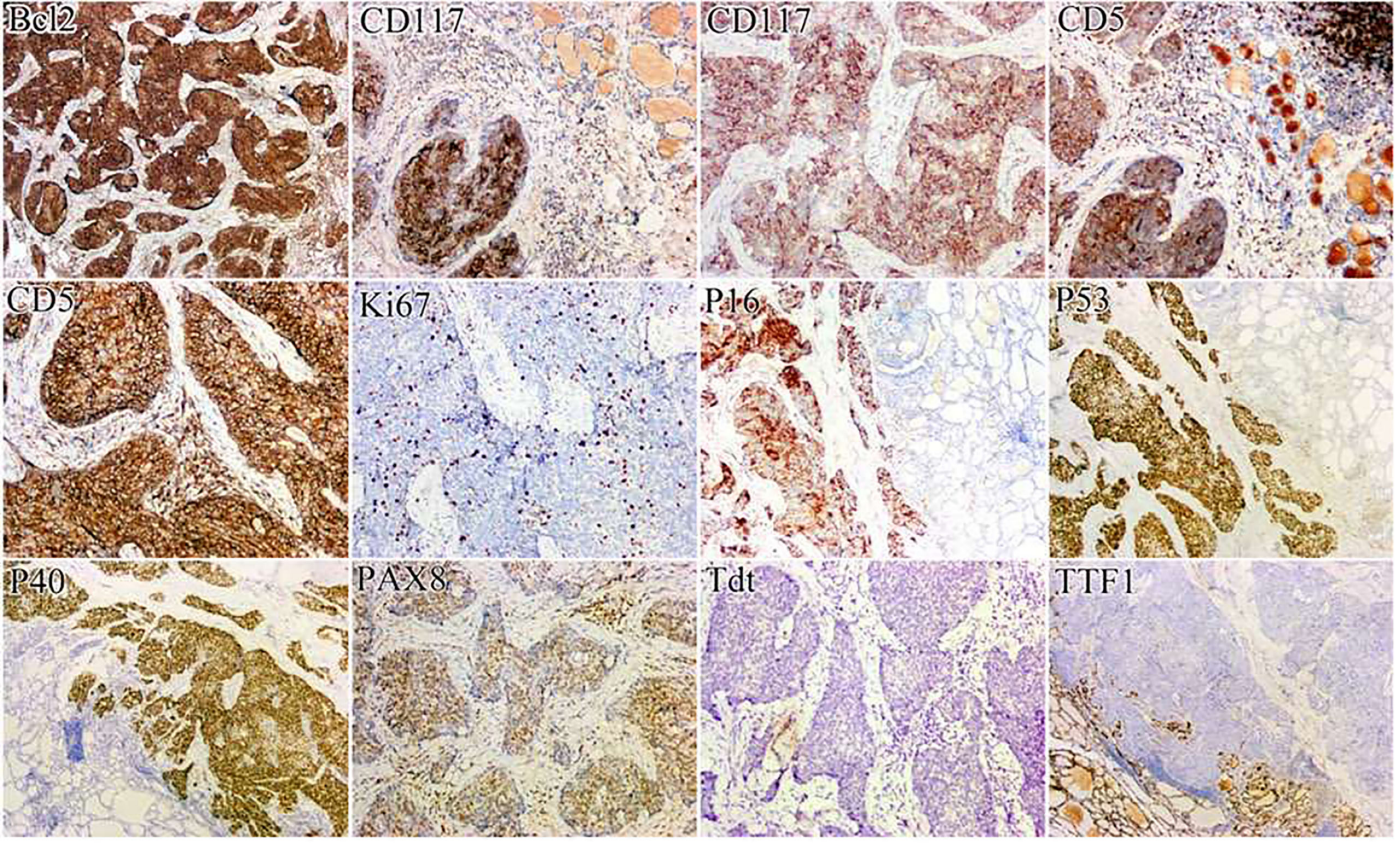
Immunohistochemically, the tumor cells were positively immunoreactive for CD5, CD117, P40, P53, Pax 8, and Bcl-2, and negatively for TTF-1.

**Figure 3:**
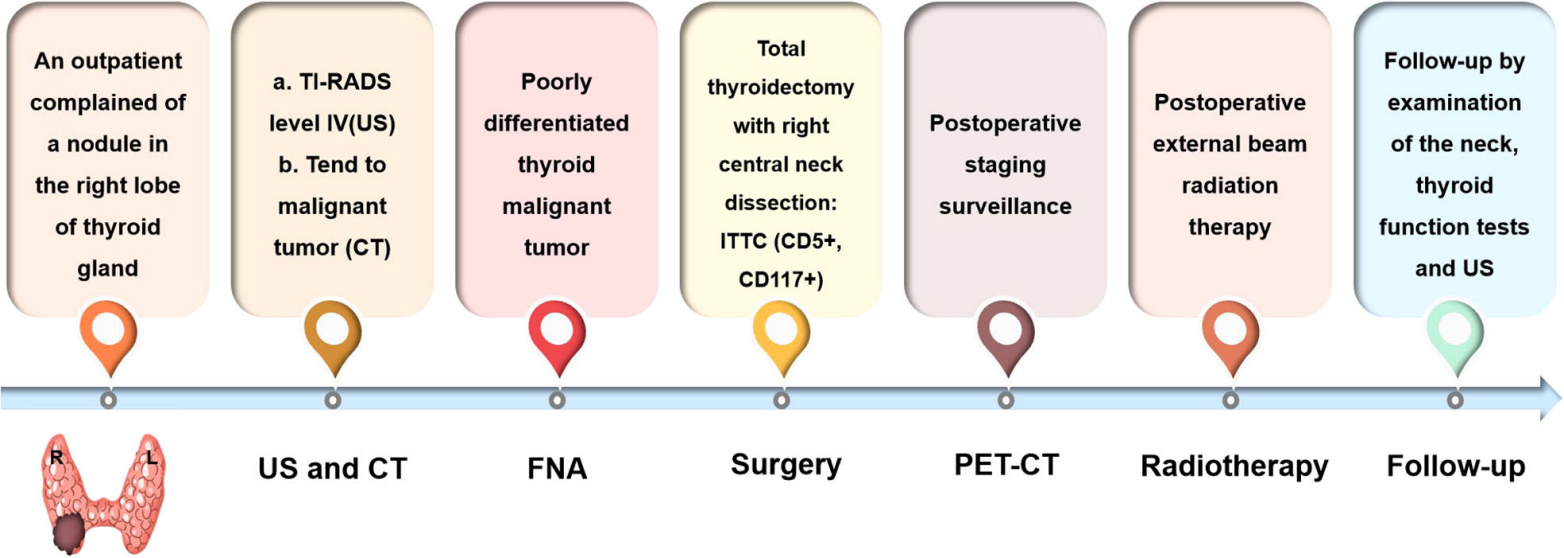
Process of diagnosis and treatment for the case.

A 41-year-old woman was referred to our hospital for further evaluation due to a thyroid nodule that was detected during a routine examination six months ago. She reported no symptoms at the time. Upon physical examination, a hard and fixed mass of 20mm in size was detected. No apparent lesions were detected in the oral cavity, nasopharynx, oropharynx, or hypopharynx. Neck ultrasonography (US) showed a solid hypoechoic nodule, sized 16mm×11mm×13mm in the middle-lower region of the right thyroid gland. The ratio of nodule longitudinal diameter to transverse diameter was less than 1. The nodule exhibited an irregular shape and unclear border, but calcification and linear hyperechoic patterns were not observed. Color Doppler sonography indicated meager blood flow within the nodule (**Figure 1**). Notably, no visibly abnormal lymph nodes were detected in the neck area. The tumor was categorized as the fourth tier of Thyroid Imaging Reporting and Data System (TI-RADS), as designated by the American College of Radiology. A comprehensive biological examination was conducted, which encompassed a serum lipid profile, blood glucose level, thyroid hormone level, antithyroglobulin antibody level, thyroglobulin level, parathyroid hormone level, calcitonin level, and tumor marker level, all of which returned normal results. Enhanced neck computed tomography (CT) indicated an 11mm sized heterogeneous, slightly enhancing mass. The local thyroid capsule was not prominently displayed. A fine-needle aspiration biopsy (FNAB) guided by US was subsequently performed, revealing smears with plump epithelioid and spindle cells, accompanied by infiltration of lymphoid cells and eosinophils.The tumor cells exhibited nuclear atypia, displaying very high nuclear to cytoplasmic ratios. The aspirate was diagnosed as a poorly differentiated malignant thyroid tumor. To obtain a definitive diagnosis, we scheduled an excisional biopsy of the mass. During the procedure under general anesthesia, the tumor, found in the middle-lower section of the right thyroid, was solid and had infiltrated a part of the sternothyroid muscle. At last, she underwent total thyroidectomy and the right central neck dissection (ND). Surgical margins were negative. Microscopically, the tumor was composed of atypical cell nests separated by dense fibrous septa with many lymphocytes and plasma cell infiltration. The neoplastic cells displayed mild-to-moderate atypia and demonstrated squamous differentiation. No histological findings were observed that indicated the presence of the typical thyroid tumors, such as papillary carcinoma, follicular carcinoma, or medullary carcinoma. Immunohistochemical analysis revealed that the tumor cells were positively reactive to CD5, CD117, CK19, P40, P53, Pax 8, and Bcl-2, and negative to thyroglobulin (Tg), thyroid transcription factor-1 (TTF-1), and calcitonin (**Figure 2**). All lymph nodes were found to be negative. Postoperative staging surveillance with PET scan was carried out, revealing no abnormal FDG uptake in distant areas, blurred fat space in the operative area, and increased uptake in the operation bed, which were considered postoperative changes. External beam radiation therapy (50.0 Gy/25 fractions) was administered to the thyroid bed and bilateral cervical lymph node area. The decision to administer adjuvant radiotherapy was grounded in institutional guidelines for primary thymus epithelial tumors (TET). The reason for this is that ITTC shared similar characteristics with TET in terms of morphology, immunohistochemistry, and genetics. Therefore, in the absence of established guidelines, the decision to apply adjuvant radiation therapy for ITTC should be based on the clinical practice guideline for primary TET. In this case, the tumor infiltrated a portion of the sternothyroid muscle, which corresponds to extraparenchymal extension of TET (equivalent to Masaoka clinical stage IIb which will be further discussed in “Discussion”). In this situation, adjuvant radiation therapy is usually indicated for improvement of local control. During the 4-year follow-up, which included physical examination of the neck, thyroid function tests, and US for the neck, she showed no signs of locally recurrent or distant disease (**Figure 3**).

## Discussion

### Clinical features

ITTC usually occurs in individuals in their fifth decade, with a male-to-female ratio of 1:1.22 (7). Clinical presentations of ITTC vary among patients, with most presenting with slowly growing neck masses that are not tender upon physical examination or ultrasound screening. However, a minority experience complaints of hoarseness due to infringement of the ipsilateral recurrent laryngeal nerve and progressive difficulty swallowing. Ge et al. (7) conducted a clinical analysis of 82 cases of ITTC and concluded that thyroid function tests showed normal results in nearly all the cases examined, indicating that ITTC does not affect thyroid function. The majority of ITTC was located in the middle-lower part of the thyroid gland, particularly in the lower pole. This observation could be explained by ITTC originating from ectopic thymic tissue or branchial pouch remnants located near or adjacent to the thyroid gland (2, 7).

### Imaging findings

Diagnosing ITTC preoperatively is difficult because the clinical manifestations and imaging results, obtained from ultrasound, CT, and MRI scans, are similar to those of other aggressive and advanced thyroid carcinomas. Therefore, a definitive diagnosis mainly depends on postsurgical pathological examination, especially immunohistochemistry studies (8). In the majority of previous reports on ultrasound examination, ITTC has been described as a solid and hypoechoic or extremely hypoechoic mass. Usually, the echo exhibits heterogeneity without cystic lesions or calcifications, which are crucial features used to diagnose SCC, papillary carcinoma, follicular carcinoma, and AC. Nevertheless, in Dong et al.’s study (9), microcalcification and/or macrocalcification were detected in three patients, suggesting that the presence of calcification could not entirely rule out the possibility of ITTC. Moreover, Yixing et al. (10) reported 11 ITTC cases, with 90% of them exhibiting linear hyperechoic patterns that can also be observed in ectopic intra-thyroidal thymus; however, this sonographic feature has not been observed in other aggressive thyroid carcinomas. It is possible that there is a correlation with the separation of fibrous connective tissue among clusters of tumor cells within the ITTC. But linear hyperechoic patternswere not observed in this case. Lesions of ITTC with abundant blood flow signals are rare. Interestingly, Stasiak et al. (11) reported the utilization of shear wave elastography (SWE) for distinguishing between intra- and extra-thyroidal ectopic thymus(IET,EET). They conducted SWE on 31 children with 53 ectopic thymuses (ETs) and calculated quantitative values of SWE parameters. The mean shear wave stiffness (SWS) of IETs (SWS_IET_) was 7.47 ± 1.93 kPa, whereas the mean SWS of the adjacent thyroid tissue was 8.66 ± 2.42 kPa. No significant distinction was found in terms of SWS_IET_ and the normal thymus SWS (SWSt) (P=0.236). The stiffness of IETs exhibited either comparable or lower values than the stiffness of the adjacent thyroid tissue. Therefore, SWE may serve as a valuable diagnostic tool in differentiating between ETs and malignant lesions. Additionally, strain elastography has demonstrated comparable differential diagnostic outcomes (12). These studies offer novel insights into ultrasound-based differential diagnosis of ITTC and other malignant thyroid tumors.

A CT scan reveals masses with soft tissue density and indistinct borders, along with rare calcification and cystic changes. These findings are not consistent with neither squamous-cell nor anaplastic carcinoma of the thyroid. A mild enhancement is observed on contrast-enhanced CT. Metastatic lymph nodes exhibit a similar appearance on CT, characterized by a low-density mass with unclear borders, no cystic formations, and slight enhancement following contrast administration. Nevertheless, MRI does not provide any diagnostic advantage (13).

### Pathological findings

FNAB is currently the recommended method for the initial pathological evaluation of thyroid nodules. Studies conducted by Ge et al. (7) and Gao et al. (14) demonstrated that FNAB can accurately diagnose masses as malignant with a range of 79.20% to 98.5%. However, the sensitivity of FNAB in diagnosing ITTC is low, ranging from 1.5% to 8.3%. Therefore, if necessary, follow-up examinations should be performed due to the high false-negative rates associated with FNAB (4). Immunohistochemistry may assist in the diagnosis of ITTC and distinguish it from other malignant thyroid neoplasms. Previous studies showed that ITTC is immunohistochemically positive for CD5 and CD117, but negative for TTF-1, Tg, and calcitonin, demonstrating that ITTC featured thymus-like differentiation rather than thyroid differentiation (15, 16). Ren et al. (17) also reported that it often shows other markers such as Pax8 (100%), p63 (100%), p53 (100%), Bcl-2 (96.42%), high-molecular-weight cytokeratin (HMWK) (100%), and EGFR (93.75%). The diffuse expression of p63 and HMWK further indicated that ITTC essentially possesses a property of the squamous cell in nature (16). Recently, it has been found that PAX8 is expressed in TET and especially overexpressed in thymus SCC. It is noteworthy that PAX8 is expressed in some types of thyroid carcinoma, such as undifferentiated or poorly differentiated cancers, which share similar morphological features with ITTC. Therefore, relying solely on PAX8 for pathologic diagnosis of ITTC is inadequate, and a combination of other markers is necessary for differential diagnosis (18). Additionally, Wang et al.’s study (19) found consistent strong expression of GLUT-1 in all cases of ITTC. This suggested that GLUT-1 can be used as a novel biomarker for ITTC and is of great diagnostic value. According to Yamazaki et al. (20), the expression of neuroendocrine markers, such as Syn and CgA, in ITTC also supported the idea that this thyroid carcinoma originates from the thymus because neuroendocrine markers have been reported to be positive in thymic carcinoma in a focal or dispersed distribution.

### Treatments

Due to the inaccuracy of preoperative examinations in diagnosing ITTC, surgery is generally preferred as the primary treatment. According to Gao et al. (14), 79.69% of analyzed cases showed infiltration of ITTC into adjacent tissues/organs, with 68.8% of cases also experiencing metastasis to regional lymph nodes. It is worth noting that patients with positive nodes had a significantly shorter survival time than those without, indicating that lymph node metastasis and extrathyroidal extension are two prognostic factors that directly affect patient survival (6, 9, 21).

### Surgery

Dong et al. (9) recommend radical surgery included thyroidectomy, resection of invaded adjacent tissues/organs, and ND was applied in cases with ITTC. Indications of total thyroidectomy are for patients with gross extrathyroidal extension, lymph node metastasis or distant metastasis. It is necessary for all ITTC tumors to perform central ND. Gao et al. (14) subgroup analysis showed that ND significantly extended the survival in patients with extrathyroidal extensions since lymph node metastasis is more likely to occur in patients with extrathyroidal extensions. Prophylactic regional ND may be an effective way to reduce the incidence of local recurrence.

### Radiotherapy

Considering similar characteristics between ITTC and TET in terms of morphology, immunohistochemistry, and genetics. Therefore, in the absence of established guidelines, patients with ITTC may also benefit from evolving therapeutic options for patients with TET. Adjuvant radiotherapy is usually advocated for patients with incompletely resected tumors and Masaoka stage III in most studies. Conversely, the effectiveness of adjuvant radiotherapy for completely resected early-stage TET has been a matter of debate, especially for stage II, which corresponds to extra-parenchymal extension of ITTC (22–28). The Masaoka clinical stage and related modified clinical staging system as follows: stage Ia, complete resection of a thymoma, macroscopically completely encapsulated and with no microscopic capsular invasion; stage Ib, complete resection of a thymoma, no microscopic capsular invasion, but with peritumoral adherences (due to fibrosis, adhesion between the tumor and adjacent structures is observed during surgery, but without microscopic evidence of capsular invasion); stage IIa, microscopic invasion into capsule; stage IIb, complete resection of a thymoma with macroscopic invasion into surrounding fatty tissue or mediastinal pleura; stage IIIa, complete resection of a thymoma with macroscopic invasion into neighboring organ (pericardium, great vessels, or lung); stage IIIb, incomplete resection or biopsy of a thymoma with macroscopic invasion into neighboring organ (pericardium, great vessels, or lung); stage IVa, complete resection of a metastatic thymoma; and stage IVb, incomplete resection of a metastatic thymoma (29–32). A systematic review and practice guideline for the management of thymoma based on the Masaoka staging system has reported that complete surgical resection alone is sufficient for the achievement of an excellent outcome in the treatment of stage I TET. The five-year overall survival rates ranged from 89% to 100%, and the local control rate approached 100%. Neither postoperative nor neoadjuvant radiotherapy is recommended for stage I disease (33). Nevertheless, according to a study by Regnard et al. (32), the recurrence rate was significantly higher among patients with peritumoral adherence (5/26 patients) compared with those without peritumoral adherence (0/109 patients) in stage I (P = 0.001). Thus, it implies that adjuvant radiotherapy, more or less, would be beneficial concerning local control in patients with peritumoral adherence in stage I of ITTC, but not to distant dissemination. In stage II of TET, the recurrence rates after resection were usually higher, reported as 11-14%, than those of stage I. The systematic review and practice guideline for the management of thymoma mentioned above advocates that routine adjuvant radiotherapy is currently not recommended for stage IIA disease (microscopic invasion into capsule), whereas it should be considered for patients with high risk, including stage IIB (complete resection of a thymoma with macroscopic invasion into surrounding fatty tissue or mediastinal pleura), close to surgical margins, WHO grade B type, tumor adherent to the pericardium and in cases of local recurrence. Meanwhile, we must also pay particular attention to the risks of acute and long-term toxicity from radiotherapy, particularly the risks of coronary artery disease and the development of secondary malignancies (33). Our case underwent postoperative radiotherapy due to involvement of the sternothyroid muscle, which corresponds to Masaoka clinical stage IIb. During the 4-year follow-up, there was no evidence of local recurrence or distant disease. Additionally, intensity-modulated radiotherapy (IMRT) which is a breakthrough of radiation technologies in the past decade has gained popularity in the treatment of head-and-neck cancers (34–36). Kong et al. (37) were the pioneers in assessing the effectiveness and safety of adjuvant IMRT after surgery for ITTC. In their study, all 14 patients (7 with lymph node metastasis and 9 with tumor extension to adjacent organs) received adjuvant IMRT, and only one patient experienced local recurrence. This finding indicated a favourable local regional control for ITTC treatment with adjuvant IMRT, while maintaining acceptable levels of toxicity. Given the rarity and typically indolent clinical progress of ITTC, a multi-institutional prospective clinical study is warranted (**Table 1**).

**Table 1:** Comparison between ITTC, primary squamous cell carcinoma of thyroid gland, differentiated thyroid cancer, and anaplastic thyroid cancer.

	ITTC (2, 7–10, 13–16, 33)	Primary squamous cell carcinoma of thyroid gland (38, 39)	Differentiated thyroid cancer (39, 40)	Anaplastic thyroid cancer (41, 42)
Epidemiology				
Morbidity	<0.15% of all thyroid cancer	0.5% of all thyroid cancer	over 95% of all thyroid cancer	4.2% of all thyroid cancer
Susceptible age	50s	70s	20-50 years	55-65 years
Sex (male to female ratio)	1:1.22	1:2.4	women representing about 25% of all patients	women representing 55–77% of all patients
Clinical features				
**Main clinical symptoms**	mostly slowly growing neck mass	rapidly increasing neck mass invading the adjacent structures with accompanying cervical lymphadenopathy	mostly slowly growing neck mass	rapidly increasing neck mass invading the adjacent structures with accompanying cervical lymphadenopathy
Imaging examinations				
Ultrasound	a. solid and hypoechoic mass b. without cystic lesions c. almost no calcification d. Possible linear hyperechoic patterns	a. large heterogeneous hypoechoic or hypoechoic mass b. possible microcalcification c. poor blood flow d. Ipsilateral lymph nodes metastasis	a. cystic necrosis and calcification b. avid enhancement	a. solid and hypoechoic mass b. irregular margins c. possible calcification d. wider than tall shape e. involvement of the cervical lymph nodes
CT	a. soft tissue density mass without calcification b. appeared enhanced following administration of contrast medium	a. large and irregularly lobulated soft tissue mass with border blurring b. prone to necrosis and hemorrhage cystic changes c. easily break through the thyroid capsule and direct invasion into adjacent organs, and/or lymph nodes metastasis d. mild to moderate enhancement	a. low or soft tissue density with cystic necrosis and/or calcification b. avid enhancement	a. mostly large, solid, and ill-defined mass frequently with necrosis, nodular calcification b. direct invasion into adjacent organs, and/or lymph node metastasis c. uneven enhancement d. degree of enhancement was relatively low
SPECT	cold nodule	cold nodule	cold nodule	cold nodule
18F-FDG PET/CT	increased uptake in the thyroid lesion	a high metabolic activity and fast growth	a. positive uptake varied from 2.2 to 3.8% b. more than half showed a focal uptake	Increased uptake in the thyroid lesion and mostly metastatic lesions
Pathology				
FNAB	the sensitivity was only 1.5% to 8.3%	/	/	/
Immunohistochemistry	a. mostly CD5 (+), CD117 (+) b. TTF-1 (-), Tg (-)	a. CK19 (+), P63 (+), CK5/6 (+), EMA (+) and PAX-8 (+) (80% and based on limited number of cases) b. TTF-1 (-), Tg (-)	mostly Tg (+) TPO (+), CD57 (+), CK19 (+), galectin3 (+), HBME1 (+)	a. mostly CK (+) and TP53 (+) b. TTF-1 (-), Tg (-) and CEA (-)
Treatment				
Surgery	first choice	first choice	first choice	first choice
Radiotherapy	for incompletely resected tumors and extraparenchymal extension of ITTC to reduce the regional recurrence rate	poorly responsive to radiotherapy	used in coordination with surgery	if tumor cannot be surgically excised
Chemotherapy	should be attempted in patients with advanced or metastatic disease	relatively resistant to chemotherapy	not suggested	a. doxorubicin plus cisplatin as radiation sensitizer b. not responsive to ^131^I therapy
Other	/	/	TSH suppression therapy	/
**Prognosis**	5- and 10-year CSS rates were 90% and 82%	2-and 10-year CSS rates were 14% and 12%	5- and 10-year CSS rates were 98 and 96%	5-year CSS rates were 5 and 15%

This symbol "/" indicates that no relevant information was found.

### Innovation and deficiency

This article presents typical and high-definition immunohistochemistry images for readers to gain a more intuitive understanding. With the complete process from first clinical visit until follow-up after adjuvant radiotherapy, surgeons can have a comprehensive grasp of the entire process of diagnosis and treatment of ITTC. Based on this case and previous literature, we suggest that routine adjuvant radiotherapy may be considered for patients with incompletely resected tumors and certain extraparenchymal extension of ITTC. Furthermore, our study provides a comprehensive overview of the advanced diagnosis, treatment, and prognosis of ITTC, as well as a comparison between ITTC, primary squamous cell carcinoma of the thyroid gland, differentiated thyroid cancer, and anaplastic thyroid cancer.

However, there are still several shortcomings in our study. Specifically, the case images did not provide sufficient detail, as we were lacking both ultrasound elastography and preoperative PET images.

## Conclusion

ITTC is an extraordinarily rare, indolent and slow-growing malignant neoplasm of the thyroid gland. It may display various clinical manifestations and it is difficult to diagnose ITTC preoperatively, but the possibility of ITTC should be warned if ultrasound detects linear hyperechoic patterns in a thyroid mass which can also be spotted in the ectopic intra-thyroidal thymus. Furthermore, elastographic features by ultrasound may provide new ideas for the differential diagnosis of ITTC and other thyroid malignant tumors. Immunohistochemically, in addition to the positive expression of CD5 and CD117 in ITTC, some new immunohistochemical markers like PAX 8, GLUT-1 had been demonstrated to give promising results for differential diagnosis. Radical resection is the fundamental treatment for ITTC, and the prognosis for this disease is favorable following surgery. In combination with this case and previous literature, routine adjuvant radiotherapy may be considered in patients with incompletely resected tumors and extraparenchymal extension of ITTC.

## Data availability statement

The original contributions presented in the study are included in the article/supplementary material. Further inquiries can be directed to the corresponding authors.

## Ethics statement

This study was performed in accordance with the principles of the Helsinki Declaration and approved by the Ethics Committee of Zhejiang Provincial People's Hospital (2021QT335). Written informed consent was obtained from the individual(s) for the publication of any potentially identifiable images or data included in this article.

## Author contributions

All authors made a significant contribution to the work reported, whether that is in the conception, study design, execution, acquisition of data, and interpretation, or in all these areas; took part in drafting, revising or critically reviewing the article; gave final approval of the version to be published; have agreed on the journal to which the article has been submitted; and agree to be accountable for all aspects of the work. AC and YD made a significant contribution to the work of collating and following the case. HH and LS designed and supervised this study. AC, CH and HH have drafted and written the manuscript. YD, LS and LZ substantially revised and critically reviewed the article. HH and AC provided the financial support. All authors contributed to the article and approved the submitted version.
